# REALITY OF INFLAMMATORY BOWEL DISEASE IN BRAZILIAN PREGNANT WOMEN (2007/2024)

**DOI:** 10.1590/S0004-2803.24612026-009

**Published:** 2026-10-26

**Authors:** Didia Bismara CURY, Liana Cristina Bismara CURY, Ana Camila MICHELETTI

**Affiliations:** 1 Clínica Scope, Centro de Doenças Inflamatórias Intestinais, Campo Grande, MS, Brasil.; 2 Universidade Anhanguera-Uniderp, Campo Grande, MS, Brasil.; 3 Universidade Federal de Mato Grosso do Sul, Instituto de Química, Campo Grande, MS, Brasil.

**Keywords:** Adverse events, biologic therapy, Crohn’s disease, pregnancy, ulcerative colitis, Eventos adversos, terapia biológica, doença de crohn, gravidez, retocolite ulcerativa

## Abstract

**Background:**

Inflammatory bowel diseases (Crohn’s and Ulcerative colitis) have been increasing alarmingly in Brazil. They affect mainly young people at reproductive age, which is extremely worrying in such a young country. To date, there´s no study that demonstrates how these diseases can affect pregnant women, like drug side effects and how off-springs of Brazilian patients evolve over time.

**Objectives:**

The study aimed to analyze the side effects of medications used by patients with Inflammatory Bowel Disease (IBD) during pregnancy; to assess the impact of disease activity on the course of pregnancy and fetal development and to monitor the development of these children.

**Methods:**

This was a longitudinal retrospective analysis of patients registered over time (2007/2024), regarding the medications used during the conception, side effects, complications during pregnancy and post-delivery. All patients were recruited at the Inflammatory Bowel Disease service at Scope Clinic, located in the Midwest region of Brazil.

**Results:**

Out of the 329 patients, 25 got pregnant (mean age 30.38 years), 48% were in endoscopic activity and 52% were in remission at conception time, and all patients maintained prescribed medication (chimeric and fully human anti-TNF agents, as well as immunosuppressive medications). Disease activity had direct influence on the babies’ weight and on the Apgar score when compared to women in remission, but despite this fact, prematurity was not observed. No undesirable drug effects were observed in both groups (mother and fetus), endorsing their safety over this population. In the postpartum follow-up over a period of two years, no abnormalities were observed.

**Conclusion:**

This study shows the importance of continuously monitoring patients with inflammatory disease and guiding them on drug maintenance and the most appropriate time for conception.

## HIGHLIGHTS

• A group that has been little studied in Brazil is pregnant women suffering from IBD, and the analysis and dissemination of information about it is extremely important.

• The active disease (IBD) at the time of conception was associated with lower baby weight and lower Apgar scores, but did not lead to prematurity or congenital malformations.

• The most used medications during conception showed no adverse effects in either the mothers or their children, reinforcing the safety of continuous medication during pregnancy.

• The study reinforces that the role of the medical community is not to discourage pregnancy in women with IBD, but rather to guide, clarify doubts and collaborate with patients’ decisions regarding the continuation of medication and the most appropriate time to conceive a new life.

## RESUMO

**Contexto:**

As Doenças inflamatórias intestinais (Doença de Crohn e Retocolite Ulcerativa) vêm aumentando de forma alarmante no Brasil. Elas afetam principalmente pessoas jovens em idade reprodutiva, o que é extremamente preocupante em um país com população majoritariamente jovem. Até o momento, não há estudos que demonstrem como essas doenças podem afetar essa população, incluindo os efeitos colaterais dos medicamentos e a evolução dos filhos de pacientes brasileiras ao longo do tempo.

**Objetivos:**

O estudo teve como objetivo analisar os efeitos colaterais das medicações utilizadas por pacientes com Doença Inflamatória Intestinal (DII) durante a gestação; avaliar o impacto da atividade da doença no curso da gravidez e no desenvolvimento fetal; e monitorar o desenvolvimento destas crianças.

**Métodos:**

Trata-se de um estudo retrospectivo longitudinal, com análise de pacientes registrados ao longo do período de 2007 a 2024, considerando as medicações utilizadas durante a concepção, efeitos colaterais, complicações durante a gestação e pós-parto. Todas as pacientes foram recrutadas do centro de Doenças Inflamatórias Intestinais do serviço da Clínica Scope, localizado no centro oeste do Brasil.

**Resultados:**

Dos 329 pacientes, 25 engravidaram (idade média de 30,38 anos); 48% estavam com atividade endoscópica da doença e 52% em remissão no momento da concepção. Todas as pacientes gravidas continuaram a terapia de base (incluindo medicamentos anti-TNF, assim como imunossupressores). A atividade da doença teve influência direta no peso dos recém-nascidos e no índice de Apgar, em comparação com as mulheres em remissão, embora não tenha sido observada prematuridade. Não foram observados efeitos adversos das medicações em nenhum dos grupos (mãe e feto), reforçando a segurança do uso desses medicamentos nessa população. No acompanhamento pós-parto, durante um período de dois anos, não foram identificadas anormalidades.

**Conclusão:**

Este estudo reforça a importância do acompanhamento contínuo de pacientes com doença inflamatória intestinal e da orientação quanto à manutenção das medicações e ao momento mais adequado para a concepção.

## INTRODUCTION

Inflammatory bowel diseases (IBD- Crohn’s and ulcerative colitis) primarily affect young patients during their reproductive age, a fact that is extremely worrying as at any given time these women may have the desire to conceive^1^. As they are chronic diseases characterized by periods of remission and exacerbation, there is a consensus that the best time to conceive is in a period of total remission, with no clinical symptoms and deep mucosa healing^2^. Thus, the doctor-patient relationship for orientation of this group is extremely important, as well as the conception planning^3^ ^,^ ^4^.

In the past, women affected by Crohn’s disease or ulcerative colitis were discouraged by doctors from getting pregnant, due to the risk of malformation that could be induced by medication or even the possibility of reactivating the disease with its withdrawal. Currently, the importance of continuous drug treatment is recognized, as well as the management of the therapies before, during and after the gestational state, however follow-up studies of mothers and their children are limited mainly to postpartum^5^ ^-^ ^7^.

Unfortunately, in Brazil this group is totally unknown. In a country of young people in which the number of cases of IBD is high, it is essential to understand their impact on Brazilian women, in relation to the behavior and evolution of the diseases during pregnancy, as well as the health of the babies during and after delivery. Furthermore, issues related to the medications used by the mothers, types of delivery, the decision they take upon lactation, what are the most common malformations, what are the complications and when they prefer to conceive, among others, were never reported^8^ ^-^ ^10^.

In general, women face doubts and uncertainties about this moment, and for those with IBD, it would be no different. Some studies have suggested that these patients must have a long and enlightening consultation on all aspects involving this topic right from the start of the treatment, and it would not be up to the doctor to discourage them from conceiving, but to clarify and make joint decisions whenever possible, advising those who wish to become pregnant to do so when the disease is clinically inactive and in advanced stages of mucosal healing^6^ ^,^ ^11^.

Undertaking a follow-up of Brazilian women has certainly become increasingly essential in a young Brazil with a growing number of Crohn’s and ulcerative colitis cases^1^ ^,^ ^9^. Herein we describe a longitudinal retrospective analysis of patients registered over time (2007/2024), with the aim of evaluating the behavior of IBD in pregnant women (evolution and complications of the disease during and after pregnancy), checking drug side effects on off-springs and assessing the health of babies before and after birth, even analyzing the type of delivery and breastfeeding period.

## METHODS

This was a longitudinal retrospective study. All patients were recruited from the Inflammatory Bowel Disease service at Scope Clinic, a private health service, located in the Midwest region of Brazil. After 17 years, an analysis of the patients’ data bank (previously cataloged) was carried out. Pregnant women were selected and their babies were analyzed at birth and after birth, as well as the evolution of mothers over time (reactivation of the disease after pregnancy). The disease activity in each patient was defined by endoscopic, histological and clinical criteria, which were previously registered in the database at the time of pregnancy. Newborn Health Assessment was performed using the Apgar method (the Apgar score was recorded in the first and in the tenth minute after birth) and birth weight and prematurity (it was considered premature children born less than 37 weeks). Postpartum evaluation was conducted for mothers and babies. For mothers it was performed during the follow-up of the patients, including routine imaging exams (colonoscopy, among others), since all patients had the disease for more than ten years. Approaches to children’s health were carried out during consultations with the mothers.

### Statement of ethics

Study approval statement: the study was approved by the ethics committee of Universidade Federal de Mato Grosso do Sul (UFMS) IRB CAAE 07702319.2.0000.0021, being the same an extension of the generated and authorized database approved by the Research Ethical Committee of the Universidade Estadual Paulista “Júlio de Mesquita Filho” (protocol number CAAE: 53006516.0.0000.5411).

Consent to participate statement: the Ethics Committee has granted exemption from obtaining informed consent of the participants as this is an analysis of a retrospective databank of de-identified data. In addition to this, the study involved patients who were over 18 years old. The patients who were under-aged were considered according to the age of diagnosis. At Clinic Scope only patients over 18 years old are accepted; the age factor was only used as an indication of the disease started.

### Data availability statement

All data generated or analyzed during this study are included in this article. Further enquiries can be directed to the corresponding author.

## RESULTS

Out of the 329 patients accompanied for IBD (2007/2024), 192 were women (58.4%), and of these, 13% got pregnant (n=25) during this period. The age at conception was between 20 to 41 years, with a mean age of 30.38 years (OR 4.87), where 56% had Crohn’s disease and 44%, ulcerative colitis. All patients included in the study underwent cesarean delivery.

During conception 48% (n=12) were in endoscopic activity and 52% in remission (n=13). Medications used during conception were azathioprine, adalimumab, infliximab, mesalazine and vedolizumab, districted according to Table 1.

**TABLE 1:** Frequency of medications used during conception.

Drug in use	n	%
Azathioprine	9	36.0
Adalimumab	7	28.0
Infliximab	5	20.0
5-aminosalicylates	1	4.0
Vedolizumab	1	4.0
None	2	8.0
Total	25	100.0

It was observed during this period that women who had active disease, remained that way until the end of pregnancy. These patients were treated with drug escalation, without any side effects, and although they remained with some sign of clinical activity, they didn´t got worse during this period. On the other hand, the disease activity directly influenced the babies’ weight (*P*<0.007, Figure 1), but not enough to lead to their prematurity. Newborns from mothers with active inflammatory disease during pregnancy presented a lower mean birth weight compared to those from mothers with inactive disease (approximately 2950 g vs 3220 g), corresponding to a mean reduction of nearly 270 g. This finding suggests a negative impact of maternal disease activity on fetal growth. The behavior of Apgar scores, used to verify babies´ health after birth, was similar to that observed for the weight (*P*<0.001, Figure 2). 

**FIGURE 1:**
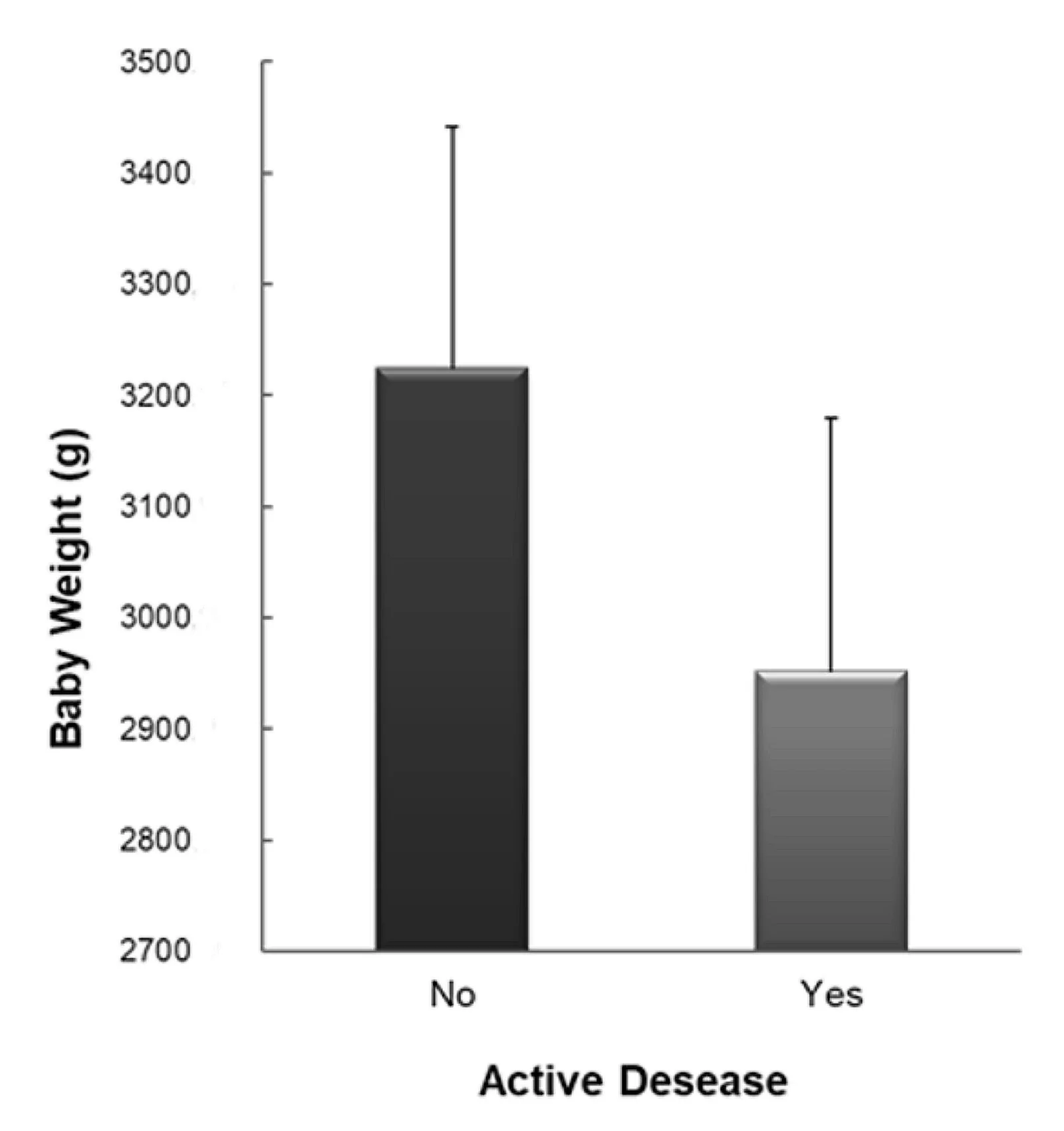
Correlation between disease activity and babies´ weight.

**FIGURE 2:**
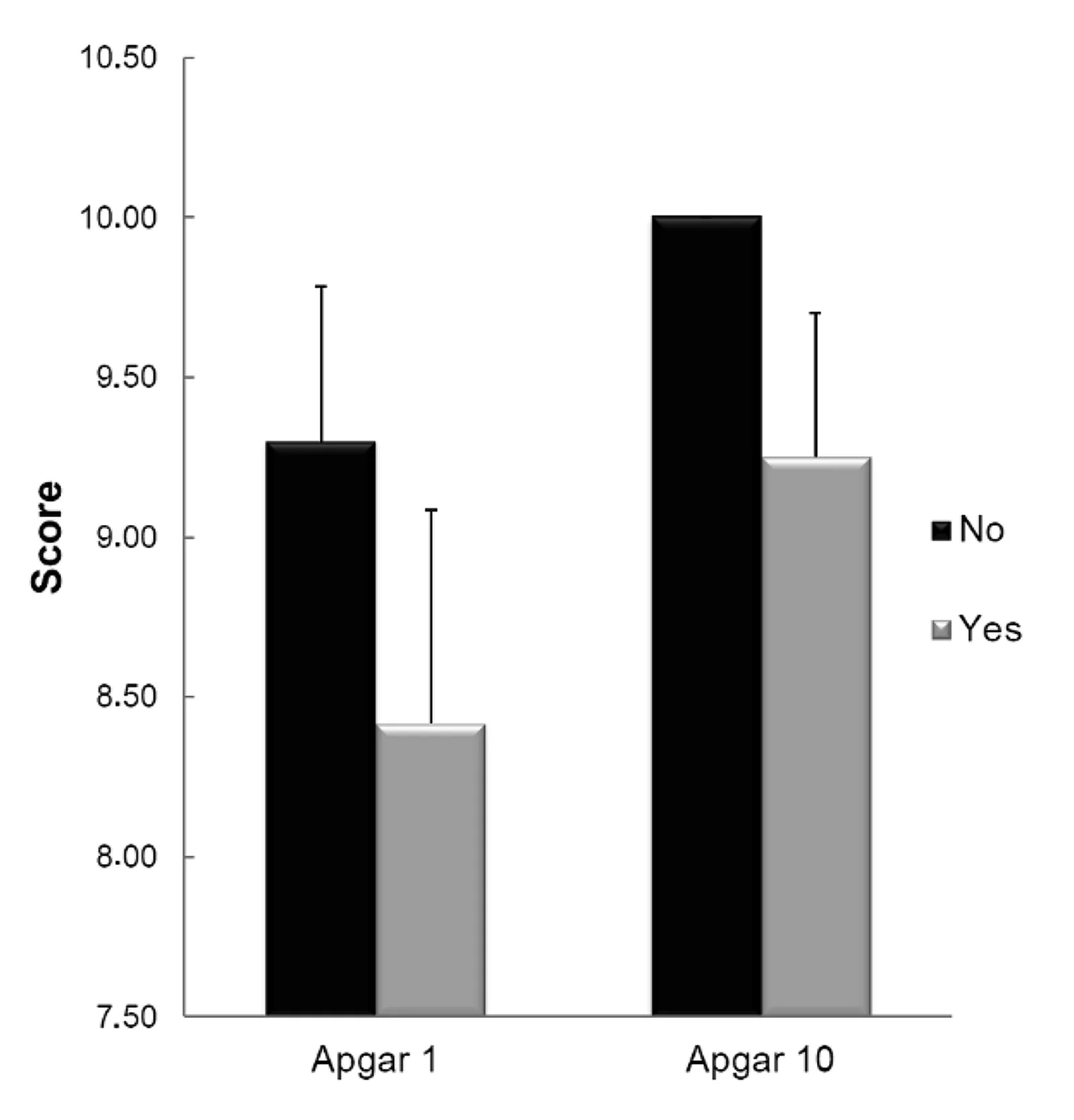
Correlation between disease activity and Apgar Score (in the first and in the tenth minute after birth).

There was no association between the degree of disease activity and babies’ weight or Apgar score. No congenital abnormalities were observed, nor were any other side effects reported, and after two years all the children were healthy. Of the pregnant women, only one reactivated the disease on the third month after delivery, her last activation episode being two years before fertilization.

## DISCUSSION

This study was developed in a Center of inflammatory bowel diseases in the city of Campo Grande, located in the Central-Western region of Brazil, with a population of high cultural level. Such data may justify the desire to conceive at a later age (30.38 years), unlike previous studies developed in public services which showed that the desire for conception occured at a younger age^9^ ^,^ ^12^ ^,^ ^13^.

Although all women were duly instructed to prepare for the best time to become pregnant from the first visit, once it is known that women in disease activity during conception tend to remain in this stage until the end of pregnancy, generating babies with low weight and prematurity^1^ ^,^ ^2^, some of them had an unplanned conception while in the active phase, when they were on treatments to achieve disease inactivity.

The disease activity was defined by imaging tests, such as colonoscopy, and continuous serum tests, associated with clinical symptoms, being diarrhea, bleeding and abdominal pain the most frequent ones. The degree of the disease activity had no influence on the babies’ weight or even Apgar scores. Curiously, even though these women had active or non-active IBD, they showed less complications than many Brazilian mothers without inflammatory disease, which reaffirms that having these conditions do not represent having a high-risk pregnancy^13^ ^-^ ^15^.

In a complementary way, the socioeconomic level and easy access to a doctor may be a more important factor than the chronic disease itself in that country. Patients in the private system, perhaps can be better cared because they have easier access to medical care than those in the public system, in addition to a better understanding of the importance of serial follow-up, nutrition, and the general care that should be provided at this moment of fundamental importance in their lives. In addition, there was no evidence of hemorrhage episodes and although they had been subjected to cesarean section, no postpartum complication was noted, unlike the study by Madeiro and co-workers^13^ in women without inflammatory disease^2^ ^,^ ^6^ ^,^ ^9^ ^,^ ^14^.

It is not yet clear whether postpartum can activate the disease in women whose disease was inactive at the time of delivery and possible causes. In our study, this relapse behavior was evidenced in only one case^10^.

No side effect of the medications was noticed in the off-springs, mothers or pregnant women who followed the same drugs from the beginning. The continuous use of these drugs during this period has been shown to avoid greater risks of complications^16^.

Unfortunately, all women decided by own choice not to breastfeed, due to the continuous therapy, for being afraid of reactivation of the disease with the withdrawal of medications or even the worsening of the disease in active women, so vaccination protocols were followed according to standards^17^.

This study has several limitations that should be considered. Its design required close and structured follow-up, including direct collaboration with an obstetrician and monthly patient monitoring. In addition, individualized counseling regarding vaccination, particularly in the context of anti-TNF therapy, was systematically provided, which may limit the reproducibility of this model in other clinical settings.

Despite these limitations, the study also presents notable strengths. The cohort consisted of highly engaged patients with a strong physician-patient relationship and consistent longitudinal follow-up. Participants received individualized guidance on vaccination, medication safety, and breastfeeding, which may have contributed to the quality and consistency of the data. Moreover, the study was conducted in a private care setting with a highly educated population, including patients with advanced academic backgrounds, which may have positively influenced adherence and data reliability.

Importantly, the continuous involvement of a dedicated obstetrician provided additional scientific and clinical support, enabling careful assessment of maternal and fetal outcomes throughout the study period. Future multicenter and prospective studies are warranted to confirm these findings and improve generalizability.

## CONCLUSION

This pioneering Brazilian study reinforces the critical role of continuous, multidisciplinary follow-up for women with inflammatory bowel disease throughout pregnancy and the postpartum period. The combination of sustained pharmacological treatment, close clinical monitoring, and psychological support represents the cornerstone of optimal maternal and neonatal outcomes. By providing original national data, this study may serve as a foundation to stimulate further research among Brazilian IBD centers, fostering a broader and more robust understanding of pregnancy in this population. Importantly, our results indicate that socioeconomic factors and access to specialized care may be more determinant of pregnancy outcomes than the presence of IBD itself, thereby challenging the traditional perception of inevitable high-risk pregnancy in these women.

## Data Availability

data not available: Research data cannot be shared (The datasets generated and analyzed during the current study are not publicly available because they contain confidential patient information and are protected by ethical and privacy regulations).

## References

[1] Allocca M, Gilardi D, Fiorino G, Furfaro F, Peyrin-biroulet L, Danese S (2018). Sexual and reproductive issues and inflammatory bowel disease: a neglected topic in men. Eur J Gastroenterol Hepatol.

[2] Cury DB, Moss AC (2014). Treatment of crohn’s disease in pregnant women: drug and multidisciplinary approaches. World J Gastroenterol.

[3] Huang V, Leung Y, Nguyen GC, Seow CH (2016). Management of inflammatory bowel disease in pregnancy: a practical approach to new guidelines. Can J Gastroenterol Hepatol.

[4] Pervez H, Usman N, Ahmed MM, Hashmi MS (2019). The impact of inflammatory bowel disease on pregnancy and the fetus: a literature review. Cureus.

[5] Auger N, Côté-daigneault J, Bilodeau-bertrand M, Arbour L (2020). Inflammatory bowel disease and risk of birth defects in offspring. J Crohns Colitis.

[6] Restellini S, Biedermann L, Hruz P, Mottet C, Moens A, Ferrante M (2020). Update on the management of inflammatory bowel disease during pregnancy and breastfeeding. Digestion.

[7] Tandon P, Govardhanam V, Leung K, Maxwell C, Huang V (2020). Systematic review with meta-analysis: risk of adverse pregnancy-related outcomes in inflammatory bowel disease. Aliment Pharmacol Ther.

[8] Lina Martins A, Volpato RA, Zago-Gomes MDP (2018). The prevalence and phenotype in brazilian patients with inflammatory bowel disease. BMC Gastroenterol.

[9] Cury DB, Oliveira R, Cury MS (2019). Inflammatory bowel diseases: time of diagnosis, environmental factors, clinical course, and management - a follow-up study in a private inflammatory bowel disease center (2003-2017). J Inflammation Res.

[10] Yu A, Friedman S, Ananthakrishnan AN (2020). Incidence and predictors of flares in the postpartum year among women with inflammatory bowel disease. Inflamm Bowel Dis.

[11] Katz S (2013). My Treatment approach to the management of ulcerative colitis. Mayo Clin Proc.

[12] Oliveira FC, Surita FG, Pinto ESJL, Cecatti JG, Parpinelli MA, Haddad SM (2014). Severe maternal morbidity and maternal near miss in the extremes of reproductive age: results from a national cross- sectional multicenter study. BMC Pregnancy Childbirth.

[13] Madeiro AP, Rufino AC, Lacerda EZG, Brasil LG (2015). Incidence and determinants of severe maternal morbidity: a transversal study in a referral hospital in Teresina, Piaui, Brazil. BMC Pregnancy Childbirth.

[14] Santana DS, Cecatti JG, Haddad SM, Parpinelli MA, Costa ML, Surita FG (2017). Severe maternal morbidity and perinatal outcomes of multiple pregnancy in the brazilian network for the surveillance of severe maternal morbidity. Int J Gynaecol Obstet.

[15] Santana DS, Silveira C, Costa ML, Souza RT, Surita FG, Souza JP (2018). WHO Multi-country survey on maternal and newborn health research network. Perinatal outcomes in twin pregnancies complicated by maternal morbidity: evidence from the who multicountry survey on maternal and newborn health. BMC Pregnancy Childbirth.

[16] Singh S, Picardo S, Seow CH (2020). Management of inflammatory bowel diseases in special populations: obese, old or obstetric. Clin Gastroenterol Hepatol.

[17] Bell SJ, Flanagan EK (2019). Updates in the management of inflammatory bowel disease during pregnancy. Med J Aust.

